# Rapid screening of SARS-CoV-2 infection: Good performance of nasopharyngeal and Nasal Mid-Turbinate swab for antigen detection among symptomatic and asymptomatic individuals

**DOI:** 10.1371/journal.pone.0266375

**Published:** 2022-04-01

**Authors:** Paola Sicilia, Gonzalo Castro, Anabella Clara Fantilli, Robertino Gierotto, Laura López, María Gabriela Barbás, María Belén Pisano, Viviana Elizabeth Ré

**Affiliations:** 1 Área Biología Molecular, Laboratorio Central, Ministerio de Salud de la Provincia de Córdoba, Córdoba, Argentina; 2 Instituto de Virología “Dr. J.M. Vanella”, Facultad de Ciencias Médicas, Universidad Nacional de Córdoba, Córdoba, Argentina; 3 Consejo Nacional de Investigaciones Científicas y Técnicas (CONICET), Buenos Aires, Argentina; 4 Área Epidemiología, Ministerio de Salud de la Provincia de Córdoba, Córdoba, Argentina; 5 Secretaría de Prevención y Promoción de la Salud, Ministerio de Salud de la Provincia de Córdoba, Córdoba, Argentina; Menzies School of Health Research, AUSTRALIA

## Abstract

Although the nasopharyngeal swab (NPS) is considered the gold standard for the diagnosis of the SARS-CoV-2 infection, the Nasal Mid-Turbinate swab (NMTS) is often used due to its higher tolerance among patients. We compared the diagnostic performance of the NPS and the NMTS for the Panbio™ COVID-19 antigen-detecting rapid diagnostic test (Ag-RDT). Two hundred and forty-three individuals were swabbed three times by healthcare professionals: a NMTS and a NPS specimen for the Ag-RDT and an oropharyngeal swab for real time RT-PCR. Forty-nine participants were RNA-SARS-CoV-2 positive by real time RT-PCR: 45 and 40 were positive by the Ag-RDT with NPS and NMTS, respectively. The overall sensitivity and specificity were 91.8% (95% CI: 83.2–100.0) and 99.5% (95% CI: 98.2–100.0) for Ag-RDT with NPS, and 81.6% (95% CI: 69.8–93.5) and 100.0% (95% CI: 99.7–100.0) for the Ag-RDT with NMTS. The Cohen’s kappa index was 0.92 (95% CI: 0.85–0.98). Among asymptomatic individuals, the Ag-RDT with both sampling techniques showed a high sensitivity [100.0% (95% CI: 95.5–100.0) with NPS; 90.9% (95% CI: 69.4–100.0) with NMTS], while the performance of the test decreased in samples with Ct≥ 30 and in patients tested after the first 7 days from symptom onset. Although the NMTS yielded a lower sensitivity compared to NPS, it might be considered a reliable alternative, as it presents greater adherence among patients, enabling scaling of antigen testing strategies, particularly in countries with under-resourced health systems.

## Introduction

The main in vitro tests used for the diagnosis of SARS-CoV-2 infection in clinical microbiology laboratories are based on the detection of viral RNA in nasopharyngeal swab (NPS) specimens. The most widely used technique is real time RT-PCR [1], a costly method that requires highly trained technical personnel, adequate infrastructure and sophisticated equipments. Therefore, it is not feasible to implement and perform it on a massive scale in most laboratories and health centers, especially in highly exposed developing countries, such as some low and middle-income regions from South America. The availability of timely, cost-effective and easy detection point-of-care (POC) diagnostic tests in testing centers is essential for an early diagnosis, which will allow the optimization of patient management and the implementation of measures to prevent further spread of the virus in the context of the COVID-19 pandemic. Thus, the use of antigen-detecting rapid diagnostic tests (Ag-RDTs) for SARS-CoV-2 has increased within the last months, as these test represent a quick and easy-to-perform alternative for virus detection [2].

The Panbio^TM^ COVID-19 Antigen Rapid Test Device (Abbott) is an in vitro Ag-RDT for the qualitative detection of SARS-CoV-2 antigen, approved through the WHO Emergency Use Listing procedure in 2020 [3]. Although the NPS specimen is considered the gold standard reference specimen type [4, 5], Nasal Mid-Turbinate swab (NMTS) sample is also frequently used, since it represents a less invasive sampling method and, therefore, more comfortable for individuals, offering new opportunities for SARS-CoV-2 testing strategies [6].

In the present diagnostic accuracy study, two professional-collected specimen types (Nasal Mid-Turbinate and Nasopharyngeal swabs) were evaluated and compared using the Panbio^TM^ COVID-19 Ag Rapid Test Device assay (Abbott).

## Materials and methods

This manufacturer-independent study took place on the 19th of February, 2021, and it enrolled 243 individuals that attended an outpatient screening center for the detection of SARS-CoV-2 infection within the framework of the COVID-19 pandemic in Córdoba, the second most populated city from Argentina. Patients were invited to voluntarily participate in this study. By that date, in Córdoba, 20.3% of adult critical care beds were occupied.

All participants showed suspected SARS-CoV-2 infection according to the local governmental testing criteria, which included adults with suggestive symptoms of COVID-19 and/or asymptomatic individuals that had a recent exposure to a SARS-CoV-2 confirmed case or were subjected to the test for other reasons (labor requirement, travel, etc.). Each individual was swabbed three times by a healthcare professional with a standardized sampling technique, following the manufacturer’s instructions [7]: 1-a NMTS sample in both nostrils for the Panbio^TM^ COVID-19 Ag Rapid Test Device (Nasal Mid-Turbinate); 2-a NPS sample for the Panbio^TM^ COVID-19 Ag Rapid Test Device (Nasopharyngeal); and 3-an oropharyngeal swab (OS) specimen for real time RT-PCR.

The clinical and epidemiological data collected for each patient was: age, sex, day from symptom onset, potential close contact with a confirmed positive COVID-19 individual within the last 14 days, symptoms [(cough/sore throat, myalgia, fever, anosmia/dysgenesis, gastrointestinal symptoms (diarrhea), and headache] (S3 Table).

Both Ag-RDTs were performed by trained personnel immediately after sample collection -following the manufacturer’s instructions [8]- and read out at 15–20 minutes.

RNA was isolated from OS samples using the MegaBio plus Virus RNA Purification Kit II on the GenePure Pro Nucleic Acid Purification System NPA-32P (Bioer). Extracted RNA was analyzed using the DisCoVery SARS-CoV-2 Nucleic Acid Detection RT-PCR Kit (Cy5/ROX) (Multiplex Real Time RT-PCR—Ap Biotech), targeting the open reading frame (ORF1-ab) gene and the nucleocapsid protein (N) gene according to the manufacturer’s instructions [9]. The cut-off cycle threshold (Ct) value was 38 for both genes, and if the Ct values of both genes were ≤38 the specimen was defined as positive.

The diagnostic performance of the antigen was assessed using sensitivity, specificity, positive predictive value (PPV) and negative predictive value (NPV). Ag-RDT sensitivity and specificity with 95% confidence intervals (95% CI) were determined relative to real time RT-PCR, as the reference standard technique. Sensitivity was evaluated for the whole study population and according to the presence of symptoms, the Ct values for N and Orf-1 genes and the days from symptom onset. Agreement between techniques was evaluated using Cohen’s kappa score; and the positive percent agreement (PPA), and negative percent agreement (NPA) between NMTS and NPS samples on the Ag-RDT (including one false-positive), were also calculated. All analyzes were performed using Epidat 3.1 [10].

Descriptive statistics were employed to summarize data. A Two-Proportion Z-Test was used to compare test sensitivities obtained from symptomatic and asymptomatic individuals, and the McNemar’s test for paired data was used for the other categories (Ct values; days from symptom onset; etc). These analyzes were performed using STATA version 14.0 (Stata Corp., College Station, TX, USA). Statistical significance was defined as p<0.05. The sample size was calculated based on a type I error of 5%, a power of 80% and an expected sensitivity in accordance with the performance data reported by the manufacturer [8]. The study was conducted according to the guidelines of the Declaration of Helsinki (1964, amended most recently in 2008) of the World Medical Association, and in accordance with specific local ethics regulations, established by the Ministry of Health of Córdoba province, Argentina.

Oral informed consent was obtained from all subjects involved in the study and from parents or guardian of minor participants (age<18). A database with their answers was registered in the informatic system of the local government. The Government of the Province of Córdoba wives the written informed consent, based on the need for rapid surveillance, which allows rapid and effective decision-making in public health.

## Results

During the research, 243 participants were included. Our study population had a median age of 32 years (3–79 years) and 46.5% (113/243) were male. In total, 51.0% (124/243) were symptomatic on the day of testing with a median duration of symptoms of 3 days (1–11 days). From the total of asymptomatic participants (119/243; 49.0%), 72.3% (86/119) had had a close contact with another patient with an RT-PCR- confirmed SARS-CoV-2 infection. Clinical and diagnostic characteristics of individuals are shown in Table 1.

**Table 1:** Characteristics of the study population.

CHARACTERISTICS	RESULTS
**Number of individuals (n)**	243
SARS-CoV-2 Positive cases by real time RT-PCR	49/243 (20.2%)
**Age (years)**	
Median (Range)	32 (3–79)
**Sex**	
Male	113 (46.5%)
**Risk Factors**	
Close contact	177 (72.8%)
**Diagnosis**	
Asymptomatic	119 (49.0%)
Symptomatic	124 (51.0%)
Cough/Sore Throat	15 (12.1%)
Headache	11 (8.9%)
Myalgia	10 (8.1%)
Fever	3 (2.4%)
Anosmia/Dysgeusia	2 (1.6%)
Diarrhea	1 (0.8%)
Unspecified	33 (26.6%)
Combination of symptoms	49 (39.5%)
**Test from symptom onset (days)**	
Median (range)	3 (1–11)
**Real time RT-PCR Results**	
**Ct values N gene**	
Mean Ct ± Standar deviation (range)	25.6 ± 3.9
**Ct values Orf-1ab gene**	
Mean Ct ± Standard deviation (range)	28.3 ± 3.9
**NMTS Ag-RDT Results**	
Positive	40/49 (81.6%)
Negative	9/49 (18.4%)
**NPS Ag-RDT Results**	
Positive	45/49 (91.8%)
Negative	4/49 (8.2%)

Forty-nine out of 243 participants were RNA-SARS-CoV-2 positive by real time RT-PCR (20.2%), of whom 38 were symptomatic (77.6%). Out of 243 individuals, 45 (18.5%) and 40 (16.4%) were positive by the Ag-RDT with NPS and NMTS, respectively (Table 2). Four specimens (1.6%) had a false-negative result with both, the NPS and NMTS, and all of them were symptomatic, with a mean Ct value of 32.6 (30.8–35.1) for the N gene and of 35.1 (33.5–37.4) for the ORF-1ab gene. One sample (0.41%) was false-positive only by the Ag-RDT with NPS. Five specimens (2.1%) were positive by the Ag-RDT with NPS but negative with NMTS, all of them had Ct values >28, and a mean Ct value of 30.7 (28.3–32.6) for the N gene and 33.6 (30.5–36.2) for the ORF-1ab gene, and 1 individual was asymptomatic. Detailed results are available in Table 2. Table 3 shows features of samples with discordant results.

**Table 2:** Agreement between the Ag-RDT with both sampling techniques and PCR for all samples, and divided into symptomatic and asymptomatic.

Overall				Symptomatic				Asymptomatic			
PCR				PCR				PCR			
+			-	+			-	+			-
**NPS Ag-RDT**	+	45	1	**NPS Ag-RDT**	+	34	1	**NPS Ag-RDT**	+	11	0
	-	4	193		-	4	85		-	0	108
**NMTS Ag-RDT**	+	40	0	**NMTS Ag-RDT**	+	30	0	**NMTS Ag-RDT**	+	10	0
	-	9	194		-	8	86		-	1	108

**Table 3:** Cases with discordant results between Ag-RDTs and real time RT-PCR.

Sample ID	Symptoms	Test after 7 days from symptom onset	Ct value real time RT-PCR		Ag-RDT NPS	Ag-RDT NMTS	Interpretation
			N	ORF-1ab			
1	Yes	No	30.8	33.5	Negative	Negative	False negative for both Ag detection.
2	Yes	No	35.1	37.4	Negative	Negative	False negative for both Ag detection.
3	No	-	32.6	36.2	Positive	Negative	False negative for Ag-NMTS detection.
4	Yes	No	28.3	30.5	Positive	Negative	False negative for Ag-NMTS detection.
5	Yes	Yes	31	34	Positive	Negative	False negative for Ag-NMTS detection.
6	Yes	Yes	29.7	32.6	Positive	Negative	False negative for Ag-NMTS detection.
7	Yes	No	32.8	35.3	Negative	Negative	False negative for both Ag detection.
8	Yes	Yes	32.2	34.8	Positive	Negative	False negative for Ag-NMTS detection.
9	Yes	Yes	31.8	34.2	Negative	Negative	False negative for both Ag detection.
10	Yes	No	-	-	Positive	Negative	False positive for Ag-NPS detection.

Sensitivity and specificity were calculated for the whole study population and according to the presence or absence of symptoms, days from symptom onset, and Ct values for the N and ORf-1ab genes. The overall sensitivity was 91.8% (95% CI: 83.2–100.0) for the Ag-RDT with NPS and 81.6% (95% CI: 69.8–93.5) with NMTS (no statistically significant difference was observed between these percentages, p = 0.063). For the above-mentioned SARS-CoV-2 prevalence (20.2%), the overall positive predictive values for the Ag-RDT with NPS and NMTS were 97.8% (95% CI: 92.5–100.0) and 100.0% (95% CI: 98.8–100.0), respectively. The overall negative predictive values were 98.0% (95% CI: 95.8–100.0) and 95.6% (95% CI: 92.5–98.6), for the Ag-RDT with NPS and NMTS, respectively.

**Fig 1:**
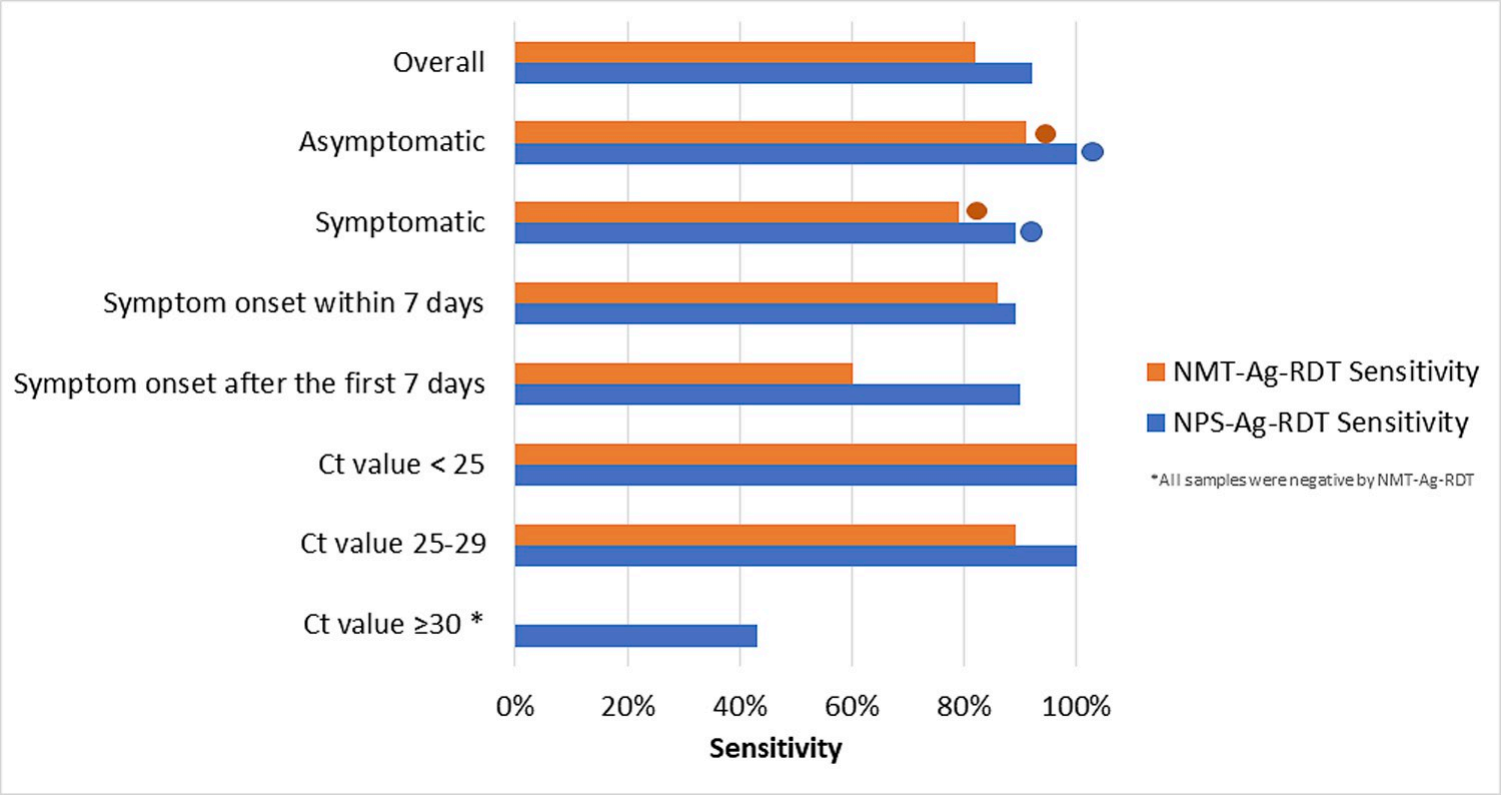
Sensitivity comparison according to the type of swabbing and the population group. *All samples in this group were negative by NMTS-Ag-RDT. Cts correspond to the N gene. Circles represent significative differences between sensitivities among symptomatic and asymptomatic individuals with both sampling techniques.

Among the categories analyzed, sensitivity percentages varied between 42.9% and 100.0% (Fig 1). The global specificity was 99.5% (95% CI: 98.2–100.0) for NPS sampling and 100.0% (95% CI: 99.7–100.0) for NMTS sampling, and were higher than 98.5% for all the categories analyzed (S1 Table). The global Cohen’s kappa index between both, NPS and NMTS, was 0.92 (95% CI: 0.85–0.98). We additionally computed the pair of agreement statistical measures: the PPA and NPA between NMTS and NPS samples on Ag-RDT, whose values were 86.96 (95% CI: 76.1–97.8) for the PPA (including one false-positive with NPS) and 100.0% (95% CI: 99.8–100.0) for the NPA.

When participants were classified in symptomatic and asymptomatic, it was observed that in symptomatic patients, Ag-RDT with NPS and NMTS sampling yielded a sensitivity of 89.5% (95% CI: 78.4–100.0) and 79.0% (95% CI: 64.7–93.2), respectively (p = 0.125) (Fig 1). Specificity was 98.8% (95% CI: 96.0–100.0) for NPS and 100.0% (95% CI: 92.4–100.0) for NMTS sampling. On the other hand, sensitivity of the Panbio test among asymptomatic individuals was 100.0% (95% CI: 95.5–100.0) for NPS and 90.9% (95% CI: 69.4–100.0) for NMTS sampling (S1 Table) (p = 1.000). The Cohen’s kappa index between the Ag-RDT with both sampling techniques was 0.90 (95% CI: 0.81–0.98) for symptomatic individuals and 0.95 (95% CI: 0.85–1.00) for the asymptomatic ones. Differences in test sensitivities between symptomatic and asymptomatic individuals were statistically significant with both sampling techniques (p value<0.001 with NPS and NMTS) (Fig 1).

Regarding Ct values for both genes, a comparison analyzes was made organizing the total of real time RT-PCR positive samples (n = 49) in sub-groups: Ct<25 (23/49); Ct between 25–30 (19/49); Ct≥30 (7/49) for the N gene. Test sensitivities for each category is detailed in S1 Table and in Fig 1. Approximately 96.0% of all viable specimens with Cts<30 for the N gene and the 100.0% of the samples with Cts<30 for the ORF-1ab gene were detected by the Ag-RDT with both specimen types. Alongside an increase in Ct values for the N gene, it was observed a reduction in the number of real time RT-PCR positive samples detected by the Ag-RDT with both sampling techniques: in specimens with Cts≥ 30, 3 out of 7 samples were positive by the Ag-RDT with the NPS and none of the 7 samples were detected by the Ag-RDT with the NMTS. Similar results were observed for the ORF-1ab gene: in specimens with Cts≥ 30, 11 out of 15 were positive by the Ag-RDT with the NPS and 6 out of 15 by the Ag-RDT with the NMTS.

When samples were stratified according to the days from symptom onset, both NPS and NMTS-Ag-RDT, showed a modest improvement in the sensitivity percentages among cases tested within 7 days from symptom onset (89.7 Vs. 90.0% for NPS Ag-RDT; and 85.7 Vs. 60.0% for NMST Ag-RDT, although these differences were not statistically significant) (Fig 1) (S1 Table).

Additionally, a double stratification of the samples was made, according to their Ct values and the presence of symptoms. Sensitivities and specificities were higher than 98.8% for the NPS-Ag-RDT, and higher than 80.0% for NMTS-Ag-RDT in samples with Cts<30, independently of the clinical status (S2 Table). A more remarkable drop in the identification of real time samples by Ag-RDT with NPS was observed in symptomatic individuals with Cts≥30 (33.3%), and no RT-PCR positive sample could be detected by the Ag-RDT with NMTS in symtomatic or asymptomatic population with Cts≥30. In order to compare our findings with previous similar studies, Table 4 shows the results obtained from the literature review regarding researches that assessed the diagnostic performance of different Ag-RDTs using nasal swab (Nasal Mid Turbinate and Anterior Nasal).

**Table 4:** Comparative studies that assessed the diagnostic performance of different instrument-free Ag-RDTs using nasal swab. Sensitivity and specificity results for nasal specimen in comparison to the reference technique were included in the table.

Reference, first author	Ag-RDT	Study location	Sample size	Study population	Sample types	SARS-CoV-2 prevalence (%)	Reference standard technique	Sensitivity (95% IC)	Specificity (95% IC)
Alqahtani et al., 2022	Panbio, Abbot	Bahrain	4183	Mildly symptomatic	NMTS and NPS	17.5	RT-PCR in NPS	82.1 (79.2–84.8)	99.1 (98.8–99.4)
Begum et al., 2021	InTec	Bangladesh	214	Symptomatic participants with known COVID-19 status	NS and NPS	52.3	RT-PCR in NS	80.0 (70.8–87.3)	97.4 (92.5–99.5)
	STANDARD Q, SD Biosensor							78.0 (68.6–85.7)	94.7 (88.9–98.0)
Cassuto et al., 2021.	Autotest COVID-VIRO	France	234	Volunteers with mild to moderate symptoms lasting less than 7 days	Self-collected ANS /NPS	13.7	RT-PCR in NPS	96.9 (83.8–99.9)	100.0 (98.2–100.0)
Chiu et al., 2021	INDICAID,PHASE Scientific International Ltd.	San Francisco, Oakland and San Fernando, United States	349	Symptomatic Populations within 5 days from symptom onset	ANS	22.8	RT-PCR	85.3 (75.6–91.6)	94.9 (91.6–96.9)
		Hong Kong	22994	Asymptomatic individuals		0.16		84.2 (69.6–92.6)	99.9 (99.9–100.0)
Homza et al., 2021	Test 1*	Karvina, Czech Republic	488	Mildly symptomatic and asymptomatic with a confirmed SARS-CoV-2 close contact	NPS/ANS (one nostril)	26.4	RT-PCR in NPS	46.5 (37.7–55.5)	99.4 (98.0–99.9)
	Test 2*		406			42.4		54.1 (46.3–61.7)	97.4 (94.5–9.1)
Kronberg et al., 2021	STANDARD Q, SD Biosensor	Copenhagen, Denmark	7074	Screening in general population with non-characteristic COVID-19 symptoms	ANS/OPS	0.9	RT-PCR in OPS	48.5	100.0
James et al., 2021	BinaxNOW, Abbot	Arkansas	2339	Symptomatic and asymptomatic clinical and non-clinical employees of an acute care Hospital	NS	6.5	RT-PCR in NS	56.6 (48.7–4.5)	99.9 (99.7–00)
Klein et al., 2021	Panbio, Abbot	Heidelberg, Germany	290	Adults with symptoms or a recent contact with a confirmed SARS-CoV-2 case	Self-collected NMTS/NPS	15.5	RT-PCR in NPS	84.4 (71.2–2.3)	99.2 (97.1–9.8)
Lindner et al., 2021 (1)	STANDARD Q, SD Biosensor	Berlin, Germany	146	Symptomatic adults	Self-collected NMTS/ Professional-collected NPS	27.4	RT-PCR in OPS/NPS	82.5 (68.1–1.3)	100.0 (96.5–00)
Lindner et al. 2021 (2)	STANDARD Q, SD Biosensor	Berlin, Germany	289	Symptomatic adults	Self-collected NMTS/Professional-collected NPS and OPS	13.5	RT-PCR in OPS/NPS	74.4 (58.9–85.4)	99.2 (97.1–99.8)
Lindner et al. 2021 (3)	STANDARD Q, SD Biosensor	Berlin, Germany	179	Symptomatic adults	Professional-collected NMTS	22.9	RT-PCR in OPS/NPS	80.5 (66.0–89.8)	98.6 (94.9–99.6)
Masiá et al., 2021	Panbio, Abbot	Spain	659	patients, either with COVID-19 signs/symptoms or asymptomatic contacts	NS (one nostril) /NPS	21.5	RT-PCR in NPS	44.7 (36.1–3.6)	100.0 (9.1–00.0)
Okoye et al., 2021	BinaxNOW, Abbot	Utah, United States	2645	Asymptomatic students	Self-collected NMTS	1.7	RT-PCR in self-collected NMTS	53.3 (39.1–67.1)	100.0 (99.9–100.0)
Pilarowski et al., 2020	BinaxNOW, Abbot	California, United States	878	Screening among symptomatic and asymptomatic individuals	ANS	3.0	RT-PCR in ANS	57.7 (6.9–76.6)	100.0 (9.6–100%)
Pollock et al., 2020	BinaxNOW, Abbot	Massachusetts, United States	2308	Symptomatic and asymptomatic individuals	ANS	12.7	RT-PCR in ANS	*77.4 (72.2–82.1)	99.4 (99.0–99.7)
Pollock et al., 2020	Access Bio CareStart	Massachusetts, United States	1498	Symptomatic and asymptomatic individuals	ANS	15.6	RT-PCR in ANS	57.7 (51.1–64.1)	98.3 (7.5–99.0)
Stohr et. Al, 2021	BD-Veritor System	Tilburg, Noord-Brabant, the Netherlands.	1595	Adults with symptoms or a recent contact with a confirmed SARS-CoV-2 case	Self-collected NMTS/ professional NPS and OPS	11.8	RT-PCR in OPS and NPS	49.1 (41.7–56.5)	99.9 (99.7–100.0)
	STANDARD Q, SD Biosensor		1606					61.5 (54.6–68.3)	99.7 (99.4–99.9)
Takeuchi et al., 2021	QuickNavi	Tsukuba, Japan.	862	Symptomatic and asymptomatic individuals	ANS/NPS	5.9	RT-PCR in NPS	72.5 (58.3–84.1)	100.0 (9.3–100)
Van der MoerenID et al., 2021	BD Veritor System	West-Brabant, the Netherlands.	352	Symptomatic adults	OPS-NS	4.8	RT-PCR in OPS and NS	94.1 (71.1–100)	100 (98.9–100)

NS: Nasal Swab; NMTS: Nasal Mid Turbinate swab; ANS: Anterior Nasal Swab; OPS: Oropharyngeal Swab

* Names not mentioned in the study

## Discussion

The overall sensitivities and specificities of the Panbio^TM^ Ag-RDT with both sampling techniques (nasopharyngeal swab and Nasal Mid-Turbinate swab) obtained during this study meet the WHO criteria for acceptable performance for SARS-CoV-2 Ag-RDTs (minimum requirements of 80% sensitivity and 97% specificity) [11].

Two previous similar studies up to date reported comparable diagnostic assessment for nasal specimen in Panbio Ag-RDT, whose results are consistent with our findings (Table 4). Klein et al. [6] performed a head-to-head comparison of the Panbio Ag-RDT with self-collected NMTS vs. professional-collected NPS samples. The overall sensitivities obtained were 84.4% with NMTS and 88.9% with NPS. In addition, the performance in our study is corroborated by Alqahtani et al. [12]. They evaluated the analytical accuracy of the Panbio Ag-RDT in nasal swab specimens in a group of patients with mild symptoms, with reference to the real time RT-PCR in NPS samplings, and the calculated sensitivity and specificity were 82.1% and 99.1%, respectively. Moreover, other studies targeting different SARS-CoV-2 Ag-RDTs, such as the Standard Q (SD Biosensor), showed similar results in the use of nasal swab sampling regarding efficacy and sensitivity [13–15] (Table 4).

On the other hand, another report by Másia et al. [16] informed that the Panbio test performance was not satisfactory for the nasal swab sampling and its sensitivity was much lower with this type of sample than with the nasopharyngeal one (44.7% vs. 60.5%, respectively). Discrepancies in diagnostic performance of this research compared to our results could be due to multiple reasons, including different clinical and epidemiological scenarios in which both studies were performed; variations in sampling techniques and handling procedures, which depends on health care personal in charge of sample collection in each study, such as different depths of insertion for nasal mid-turbinate swab and swabbing one or both nostrils [17]), inadequate quality of the sample collected, etc. Furthermore, because the readout of this type of assays is by visual inspection, results may be subjective, especially when bands are faint or partial. Additionally, viral kinetics in patients and the moment of the infection that they were swabbed can also be factors that contribute to discrepancies in sensitivities obtained in both studies.

SARS-CoV-2 positive samples by real time RT-PCR that were not detected by the antigen test, presented higher Ct values than those that were positive by the antigen tests with both types of specimen collection, indicating that viral load would be crucial to obtaining concordant results. Besides, when samples were divided into sub-groups according to the Ct values, all samples with RT-PCR Ct-values <25 were detectable by the Panbio Ag-RDT with both sampling techniques. Furthermore, it yielded a greater number of discordant results among patients with Ct≥ 30, consistent with findings of previous studies that assessed the Abbott Ag-RDT in different specimen types, but also with reports of commercial Ag-RDTs from other suppliers [6, 7, 12, 15, 16, 18–28]. These results could also be observed when a double stratification of the samples was performed (according to their Ct values and the presence of symptoms) (S2 Table). There is a substantial drop in sensitivity among the group of symptomatic and asymptomatic individuals with Ct≥30. However, it is important to remark that a grvowing body of evidence indicates that subjects with low viral load testing SARS-CoV-2 positive by RT-PCR and negative by Ag-RDTs have a limited capacity for effective transmission [20, 29].

Information with reference to subjects with asymptomatic SARS-CoV-2 infections is relevant because these individuals have played an important role in the pandemic, comprising a significant portion of the infected population and having a substantial impact on the spreading of this virus (32). Contrary to other similar researches that assessed the Panbio Ag-RDT diagnostic performance in asymptomatic individuals [16, 19, 22, 28, 30–32], our results yielded significantly higher sensitivity among this population with NPS and NMTS sampling techniques. One of the possible reasons that could explain this is that in our study the proportion of participants with Ct values ≥30 was higher in symptomatic than in asymptomatic participants. Besides, all samples of the latter group, except one (with a Ct value of 32.6, the only one with a false positive NMTS Ag-RDT result), are within the range of moderate Ct values (25–29; with a mean Ct of 27.5 for the N gene) and were detected by the rapid antigen test with both sampling techniques. Increasing trend of Panbio test sensitivity in asymptomatic participants with lower Ct values was also observed in previous reports [28, 32, 33]. Alemany et al., (2021) revealed that in their study the Panbio Ag-test sensitivity increased with Cts< 25 and < 30 (100.0% and 98.6%, respectively). Torres et al., [32] also found that the Panbio sensitivity in asymptomatic individuals was directly related to the magnitude of SARS-CoV-2 RNA load in NPS specimens. Another previous recent study by Winkel et al. [33] assessed the performance of the Panbio Ag Test in NPS samples from asymptomatic individuals, and sensitivity ranged from 80.0% to 86.7%, similar to our results. Comparable findings have also been documented in previous studies of a wide variety of different SARS-CoV-2 Ag-RDTs [16, 23, 28, 30, 34–37]. The higher sensitivity of the Panbio Ag-RDT in samples with low Ct values, irrespective of the presence of symptoms, indicates that the test is particularly suitable for identifying individuals who are contagious, and can have a high diagnostic yield for transmission relevant infections with limited false positives. Thus, it can be an appropriate epidemiological surveillance tool for universal screening [16, 23, 34, 35].

Viral load kinetics have been confirmed to be largely similar in asymptomatic and symptomatic patients, and high viral loads are expected to be found in asymptomatic subjects during early infection after exposure with close contacts [18, 33, 36, 38, 39]. Information regarding the establishment of the optimal timeframe for upper respiratory tract collection after exposure to the index case seems crucial to accurately determine the sensitivity of the test [19, 33, 34]. Nevertheless, as a limitation of our study, these data was unavailable. Furthermore, the clinical course of asymptomatic cases could not be followed, and information regarding possible posterior development of symptoms later from diagnosis could not be obtained. Hence, we can not rule out that asymptomatic individuals had actually been tested in the presymptomatic window.

Rapid antigen tests can reliably detect symptomatic SARS-CoV-2 infections in the early phase of disease, within the 7 days from symptom onset, thereby identifying the most contagious persons [31]. In this sense, our results showed a good performance for the Ag test with both sampling techniques in this population group (sensitivities≥ 85.7%). However, the sensitivity was considerably lower (although not statistically significant) when the Ag-RDT with NMTS was performed after the first 7 days from symptom onset, so the diagnostic interpretation in this group of patients should be cautious.

The use of NMTS yielded a lower sensitivity in comparison to NPS, although not statistically significant. However, other variables would counterbalance these differences in the diagnostic performance, and the use of NMTS could prove to be a viable alternative to the reference sampling method: while nasopharyngeal swab is considered more invasive and uncomfortable [27, 40], nasal sampling is of higher tolerance for patients [6], increasing adherence and acceptability of a greater number of individuals, which may improve and maximize the capacity to managing and tracing COVID-19 cases and controlling new outbreaks. Moreover, this technique can also be used for the self-sampling method, which allows more widespread and frequent testing.

Moreover, low and middle-income countries, especially during complex epidemio-logical scenarios, might face socioeconomic insufficiencies and lack of public investment in extensive laboratory structures or highly skilled healthcare professionals to implement molecular techniques. In these regions, with scarce laboratory instrumentations or with real time RT-PCR assays insufficiently available, Ag-RDTs are a highly considerable alternative for cost-reduction and large scales test strategies [22, 24, 30, 41]. In terms of operability and efficiency, these tests are easy-to-administer, allowing performance in decentralized settings and with independence from laboratory facilities. Additionally, these devices are also simple to perform and to read, cost-effective and expeditious, incrementing the pace of testing, identifying and treating people for COVID-19 at the POC.

## Conclusions

To conclude, our findings corroborate and confirm the increasing evidence base supporting the use of alternative sampling method, such as Nasal Mid-Turbinate swab, in the context of mass screening strategy during the SARS-CoV-2 pandemic, especially in developing countries or regions with under-resourced health systems. The nasopharyngeal sampling method is often uncomfortable and invasive for patients. Our findings reveal that Ag-RDTs with NMTS specimens might be recognized as a reasonable, valuable and reliable diagnostic tool for widespread utilization, particularly among individuals in the first stage of disease with high viral loads. However, these tests should be interpreted cautiously depending on the clinical and epidemiological context, and they might be used in addition to real time RT-PCR as part of the testing strategies for COVID- 19.

## Supporting information

S1 Table Overall diagnostic performance of the Panbio COVID-19 Ag test and performance according to the presence of symptoms, the Ct values (for the N and ORF-1ab genes) and days from onset of symptoms. (DOCX)

S2 Table Diagnostic performance of NPS and NMTS Ag-RDTs according to the presence of symptoms and Ct values for the N gene. (DOCX)

S3 Table Patient data. (XLSX)
