# Supplementary material for: Rapid screening of SARS-CoV-2 infection: Good performance of nasopharyngeal and Nasal Mid-Turbinate swab for antigen detection among symptomatic and asymptomatic individuals
Source: PLoS One. 2022 Apr 1;17(4):e0266375. doi: 10.1371/journal.pone.0266375 (PMC8986327; doi:10.1371/journal.pone.0266375)
Supplement: S1 Table — (DOCX) [file pone.0266375.s001.docx]

| **Group** | **N** | **Ag-RDT-NPS Sensitivity**  **%**  **(95% CI)** | **Ag-RDT-NMTS Sensitivity**  **%**  **(95% CI)** | **Ag-RDT-NPS Specificity**  **%**  **(95% CI)** | **Ag-RDT-NMTS Specificity**  **%**  **(95% CI)** | **Ag-RDT-NPS**  **PPV***  **%**  **(95% CI)** | **Ag-RDT-NMTS**  **PPV***  **%**  **(95% CI)** | **Ag-RDT-NPS**  **NPV***  **%**  **(95% CI)** | **Ag-RDT-NMTS**  **NPV***  **%**  **(95% CI)** |
| --- | --- | --- | --- | --- | --- | --- | --- | --- | --- |
| Ct value for N gene < 25 | 23 | 100.0  (97.8-100.0) | 100.0  (97.8-100.0) | 99.5  (98.2–100.0) | 100.0  (99.7- 100.0) | 95.8  (85.8-100.0) | 100.0  (97.8-100.0) | 100.0  (99.7-100.0) | 100.0  (99.7-100.0) |
| Ct value for N gene 25-29 | 19 | 100.0  (97.4-100.0) | 89.5  (73.0-100.0) | 99.5  (98.2–100.0) | 100.0  (99.7–100.0) | 95.0  (83.0-100.0) | 100.0  (97.1-100.0) | 100.0  (99.7-100.0) | 99.0  (97.3-100.0) |
| Ct value for N gene ≥30 | 7 | 42.9  (0.0-86.7) | N/A | 99.5  (98.2–100.0) | N/A | 75.0  (20.1-100.0) | N/A | 98.0  (95.8-100.0) | N/A |
| Ct value for Orf-1ab gene < 27 | 20 | 100.0  (97.5–100.0) | 100.0  (97.5–100.0) | 99.5  (98.2–100.0) | 100.0  (99.7–100.0) | 95.2  (83.8-100.0) | 100.0  (97.5–100.0) | 100.0  (99.7–100.0) | 100.0  (99.7 – 100.0) |
| Ct value for Orf-1ab gene 27-29 | 14 | 100.0  (96.4–100.0) | 100.0  (96.4–100.0) | 99.5  (98.2–100.0) | 100.0  (99.7–100.0) | 93.3  (77.4–100.0) | 100.0  (96.4–100.0) | 100.0  (99.7–100.0) | 100.0  (99.7–100.0) |
| Ct value for Orf-1ab gene ≥30 | 15 | 73.3  (47.6–99.1) | 40.0  (11.9–68.1) | 99.5  (98.2–100.0) | 100.0  (99.7–100.0) | 91.7  (71.9–100.0) | 100.0  (91.7–100.0) | 98.0  (95.8–100.0) | 95.6  (92.5 – 98.6) |
| Test within 7 days from symptom onset | 28 | 89.3  (76.0-100.0) | 85.7  (71.0-100.0) | 98.7  (95.6-100.0) | 100.0  (99.7–100.0) | 96.2  (86.8-100.0) | 100.0  (97.9-100.0) | 96.3  (91.6-100.0) | 95.2  (90.0-100.0) |
| Test after 7 days from symptom onset | 10 | 90.0  (66.4-100.0) | 60.0  (24.6-95.4) | 100.0  (92.9-100.0) | 100.0  (92.9 – 100.0) | 100.0  (94.4-100.0) | 100.0  (91.7-100.0) | 87.5  (58.3-100.0) | 63.6  (30.7-96.6) |
| Symptomatic | 124 | 89.5  (78.4-100.0) | 79.0  (64.7-93.2) | 98.84  (96.0-100.0) | 100.0  (92.4- 100.0) | 97.1  (90.2-100.0) | 100.0  (98.3-100.0) | 95.5  (90.6-100.0) | 91.5  (85.3-97.7) |
| Asymptomatic | 119 | 100.0  (95.5- 100.0) | 90.9  (69.4-100.0) | 100.0  (99.5–100.0) | 100.0  (99.5- 100.0) | 100.0  (95.5-100.0) | 100.0  (95.0-100.0) | 100.0  (99.5-100.0) | 99.1  (96.8-100.0) |
| Overall | 243 | 91.8  (83.2-100.0) | 81.6  (69.8-93.5) | 99.5  (98.2-100.0) | 100.0  (99.7–100.0) | 97.8  (92.5-100.0) | 100.0  (98.8-100.0) | 98.0  (95.8-100.0) | 95.6  (92.5-98.6) |

**S1 Table. Overall diagnostic performance of the Panbio COVID-19 Ag test and performance according to the presence of symptoms, the Ct values (for the N and ORF-1ab genes) and days from symptom onset**.

*PPV: positive predictive value; NPV: negative predictive value
