# Supplementary material for: Rapid screening of SARS-CoV-2 infection: Good performance of nasopharyngeal and Nasal Mid-Turbinate swab for antigen detection among symptomatic and asymptomatic individuals
Source: PLoS One. 2022 Apr 1;17(4):e0266375. doi: 10.1371/journal.pone.0266375 (PMC8986327; doi:10.1371/journal.pone.0266375)
Supplement: S2 Table — (DOCX) [file pone.0266375.s002.docx]

**S2 Table. Diagnostic performance of NPS and NMTS Ag-RDTs according to the presence of symptoms and Ct values for the N gene**.

|  |  | **Symptomatic individuals** | | | **Asymptomatic individuals** | | |
| --- | --- | --- | --- | --- | --- | --- | --- |
| **NPS**  **Ag-RDT** | **N gene Ct values** | **Sensitivity % (95% CI)** | **Specificity % (95% CI)** | | **Sensitivity % (95% CI)** | | **Specificity %(95% CI)** |
|  | < 25 | 100.0  (97.7-100.0) | 98.8  (96.0-100.0) | | 100.0  (50.0-100.0) | 100.0  (99.5-100.0) | |
|  | 25-29 | 100.0  (95.0-100.0) | 98.8  (96.0-100.0) | | 100.0  (99.4-100.0) | 100.0  (99.5-100.0) | |
|  | ≥ 30 | 33.3  (0.0-79.4) | 98.8  (96.0-100.0) | | 100  (50.0-100.0) | 100.0  (99.5-100.0) | |
| **NMTS Ag-RDT** | **N gene Ct values** |  | |  |  |  | |
|  | < 25 | 100.0  (97.7-100.0) | 100.0  (99.4-100.0) | | 100.0  (50.0-100.0) | 100.0  (99.5-100.0) | |
|  | 25-29 | 80.0  (50.2-100.0) | 100.0  (99.4-100.0) | | 100.0  (99.4-100.0) | 100.0  (99.5-100.0) | |
|  | ≥ 30 | N/A | N/A | | N/A | N/A | |
|  |  |  |  | |  |  | |

*N/A: not applicable due to no sample of these categories could be detected by the Ag-RDT.
